# Advancing the One Health Framework in EU Plant Protection Product Regulation: Challenges and Opportunities

**DOI:** 10.3390/jox15060200

**Published:** 2025-12-01

**Authors:** Maura Calliera, Ettore Capri, Nicoleta Alina Suciu, Marco Trevisan

**Affiliations:** 1 Opera Research Centre, Università Cattolica del Sacro Cuore, 29122 Piacenza, Italy; ettore.capri@unicatt.it; 2 Department for Sustainable Food Process (DISTAS), Università Cattolica del Sacro Cuore, 29122 Piacenza, Italy; nicoleta.suciu@unicatt.it (N.A.S.); marco.trevisan@unicatt.it (M.T.)

**Keywords:** One Health, plant protection products, European regulatory framework, risk evaluation

## Abstract

This paper explores the evolving integration of the One Health framework into the European regulatory landscape for plant protection products, focusing on key scientific and procedural challenges. The analysis addresses three fundamental components of risk evaluation—regulatory complexity, hazard identification, and characterisation—and exposure assessment, while providing an up-to-date overview of emerging policies and challenges affecting the sustainable use of plant protection products in Europe. Addressing these issues requires interdisciplinary collaboration among toxicologists, epidemiologists, ecologists, regulatory authorities, industry stakeholders, and public health experts, working synergistically to tackle complex risks. It emphasises that transitioning to more sustainable and resilient agricultural systems in line with One Health principles requires critically reviewing existing policies. By integrating evolving scientific knowledge with communication and agricultural production needs across diverse European contexts, this approach offers valuable insights to inform future policy development and risk management innovation.

## 1. Introduction

The “One Health” approach is based on understanding that human, animal, and environmental health are interconnected. It promotes collaboration between scientific disciplines and sectors such as food safety, environmental risk management, and combatting antimicrobial resistance [1].

European (ECDC, ECHA, EEA, EFSA, EMA) and international (FAO, OIE, UNEP, WHO) agencies work together to address global health challenges from a transdisciplinary perspective. This approach is also crucial for the achievement of the UN Sustainable Development Goals, particularly those related to health, environment, and partnerships [2,3].

The European Union has some of the most advanced and stringent legislation on food safety and environmental protection. Among the emerging priorities, reducing the use of Plant Protection products (PPPs) is an objective shared by many institutions, partly in response to the growing public awareness of the associated risks. However, risk assessment and risk management remain a central issue, especially in light of the new challenges posed by climate change, biodiversity loss, and the need to ensure global food security. By recognising the interdependence of human, animal, plant, and environmental health, One Health promotes integrative and sustainable strategies that jointly mitigate climate impacts while preserving ecosystem integrity [4]. For example, adopting sustainable agricultural practices aligned with One Health can enhance ecosystem resilience, reduce greenhouse gas emissions, and safeguard biodiversity. Furthermore, coordinated disease and pest surveillance that integrates data from human, animal, plant health, and environmental monitoring, enables early detection and response to emerging threats exacerbated by climate change, helping to mitigate risks holistically.

Risk analysis is a scientific and objective process aimed at estimating the likelihood of adverse effects on human health and the environment from exposure to xenobiotics. It involves the definition of a safe use dose and involves a multidisciplinary community of experts, constituting one of the pillars of European regulation. [5] However, critical issues persist that limit its effectiveness and full adherence to “One Health” principles. The ambitious objective of reducing reliance on PPPs requires more than top-down mandates; it demands leveraging the specialised expertise and practical knowledge of all stakeholders and allowing adequate time for adaptation across diverse production contexts. The policy shift in Sri Lanka—which started in 2019 and was applied in 2021, forcing a rapid transition to organic farming—led to a severe 20% drop in rice production, accompanied by economic turmoil and increased poverty among farmers. Agronomists had warned that the swift implementation without sufficient preparation or socio-economic safeguards risked undermining food security and rural livelihoods [6,7]. This experience underscores the need for carefully planned, context-sensitive strategies that balance production demands with risk management and include inclusive stakeholder engagement to support resilient food systems. While the goal of achieving a 50% reduction in pesticide use aligns with sustainability and One Health principles, it involves complex trade-offs relating to agricultural productivity, economic stability, and food security.

The implementation of the “One Health” approach in the regulation and management of PPPs in Europe is therefore a complex but unavoidable challenge for the protection of public health and the environment. A critical analysis of available policies and knowledge is essential to guide the transition towards more sustainable and resilient agricultural systems [8].

This paper critically examines the key challenges and limitations in the risk evaluation process of plant protection products (PPPs), beginning with the regulatory framework and emphasising the challenges and opportunities associated with integrating the “One Health” principle.

## 2. Materials and Methods

*Study Design and Scope.* The study follows a qualitative narrative synthesis approach aimed at critically evaluating regulatory complexity, scientific advancements in hazard identification and exposure assessment, risk characterisation methodologies, and risk communication strategies. The scope includes evaluation of EU legislation; scientific assessment process challenges, including innovative methodologies; and stakeholder engagement within a One Health context. The methodology is grounded in a structured literature and policy review, synthesising scientific, regulatory, and procedural insights from multiple authoritative sources. References published as articles, book chapters, conference proceeding, systematic review, technical reports, and project website documents were screened to ensure the relevancy with respect to the research aim and after using screening criteria as follows: to be relevant at International and European level; to be the results of a project committed by European or international and well-recognised entity such as, for example, FAO, OECD, EFSA; elements of the overall PPPs risk evaluation procedural and policy process; and relevant and/or updated to respect the on-going debate on PPPs use, sustainability, and the One Health framework.

Data were systematically collected from the following:

- Official European regulatory documents and directives, including Regulation (EC) No 178/2002 [9], Regulation (EC) No 1107/2009 [10], Sustainable Use Directive 2009/128/EC [11], and associated EU policy reforms (e.g., REFIT, Green Deal).
- Reports, guidance, and scientific publications from relevant EU agencies: Science Advice for Policy by European Academies (SAPEA), EFSA, ECHA, ECDC, and EMA.
- International health and environmental organisations such as FAO, WHO, and UNEP.
- Peer-reviewed articles and pilot project case studies.

*Analytical Framework*. The analysis was organised into thematic sections reflecting the critical pillars of PPP risk evaluation and communication under the One Health paradigm: regulatory framework complexity (Section 3.1), scientific methodologies for hazard and exposure evaluation, risk characterisation challenges (from Section 3.2, Section 3.3 and Section 3.4), communication strategies incorporating public and stakeholder perceptions (Section 3.5), and trust-building in chemical risk governance (Section 3.6).

Cross-references between legislative texts, scientific findings, and implementation case studies were employed to identify gaps, inconsistencies, and future opportunities. Emphasis was placed on the bidirectional interaction between risk assessors, risk managers, and societal actors, reflecting the complexity of One Health operationalisation.

## 3. Results

**Figure 1:**
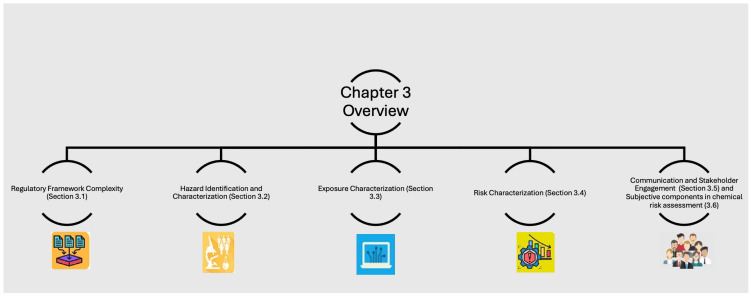
Flow of information, main steps, and key topics discussed in Section 3.

To facilitate readers’ comprehension of the logical progression and thematic structure of Section 3, Figure 1 presents a flowchart summarising the main steps and topics discussed, starting from regulatory framework complexity to risk communication and subjective components in the chemical risk assessment.

### 3.1. Complexity of the Legislative Framework

The European Union has developed a robust legislative framework aimed at jointly protecting human, animal, and environmental health. EU chemical legislation has developed over time through the adoption of specific directives and regulations, differentiated according to market types and application sectors. Thus, biocides, industrial chemicals, plant protection products, and human and veterinary medicines are regulated independently. Regulation (EC) No 178/2002 established overarching food safety principles, including risk analysis and transparency, laying the foundation for harmonised pesticide evaluation across the EU. Regulation (EC) No 1107/2009 introduced stricter risk assessment protocols, enhanced transparency and precautionary principles, and explicit criteria for approval, focusing on minimising risks to humans, animals, and ecosystems. The Sustainable Use Directive (2009/128/EC) complements product regulation by promoting integrated pest management (IPM) and reducing pesticide reliance, aiming to balance agricultural productivity with health and environmental sustainability.

This section presents the complex, multilayered European legislative framework governing plant protection products, highlighting how this regulatory complexity poses both challenges and opportunities for effectively implementing a coherent One Health approach.

#### 3.1.1. Regulatory Framework for Plant Protection Products

PPPs, commonly known also as “pesticide,” fall within the broader framework of European food legislation. In response to several food incidents in the late 1990s [12], the European Parliament and the Council adopted Regulation (EC) No 178/2002, which lays down the general principles and requirements of food law. This regulation covers all stages of the food chain: from primary production to processing, storage, transport, and retail. The main objective is to ensure a high level of protection of human and animal health, based on a scientific approach that is based on risk analysis. This analysis assesses the likelihood and severity of effects resulting from the presence of hazards in food or feed. The regulation also sets out principles of transparency, independence, and cooperation among authorities involved in food safety, and includes provisions for rapid alert systems and emergency measures in food safety crises [13]. A key feature of this regulation is the establishment of the European Food Safety Authority (EFSA), which provides independent scientific advice and risk assessments to support risk management decisions by the European Commission and Member States. EFSA plays the central role in scientific risk assessment, while the European Commission is responsible for risk management, taking the necessary preventive and control measures [14]. The risk assessment methodologies are continuously updated to address the evolving challenges, such as cumulative risk assessment, transparency, and emerging contaminants. EFSA collaborates closely with national agencies to ensure scientific thoroughness and policy relevance. This framework is structured through a combination of horizontal general rules and vertical specific substance regulations, which together form a harmonised and cooperative system across Member States [15]. Before substances such as food additives, allergens, genetically modified organisms, novel foods (e.g., insects), and PPPs can be used, they must undergo rigorous safety assessments and be authorised according to harmonised European regulations [16].

The plant protection product regulation (Regulation (EC) No 1107/2009) complements this with rigorous risk assessment protocols, ensuring only substances meeting strict safety criteria are approved, incorporating precautionary principles, environmental and operator protections, and periodic re-evaluation. Together, these regulations form a robust legislative framework for chemical food safety and PPPs in the EU, and support the broader One Health approach by addressing chemical food safety risks throughout the entire food production chain, from primary production to consumption, thus safeguarding human health while promoting sustainable agricultural practices.

#### 3.1.2. Evaluation and Authorisation of Plant Protection Products (PPPs) and Risk Management

Regulation (EC) No 1107/2009 primarily regulates PPPs, setting strict criteria to ensure that active substances have no adverse effects on human or animal health or the environment [17]. Currently, the evaluation process for the active substances contained in PPPs involves a dual-level system: at the EU level, the European Food Safety Authority (EFSA), in cooperation with Member States, carries out a peer review of risk assessments and drafts conclusions on active substances, which are then approved or rejected at the Union level by the European Commission. Concurrently, Member States perform evaluations and authorisation of individual PPPs at the national level, based on the approved active substances and considering national agricultural and environmental contexts [18].

This system allows for rigorous, centralised scientific peer review of substances, ensuring high safety and environmental standards; however, it also introduces variability in the implementation of risk management measures, due to differing national conditions, agricultural practices, and regulatory interpretations. Harmonisation remains both possible and essential within the One Health context in other critical areas, such as scientific risk assessment methodologies and standardised data requirements. In summary, while practical regulatory implementation adapts to local contexts, harmonisation at the scientific, methodological, and procedural levels is vital to integrate One Health principles effectively across the EU PPP regulatory system [19].

On the other side, risk management is also regulated during the marketing and use of PPPs by an extensive body of legislation. Directive 2009/128/EC on the sustainable use of pesticides (SUD) is an important tool to promote responsible use and reduce environmental and health risks. The SUD represents a significant step towards One Health by advocating integrated pest management and reduced PPP reliance, contributing to ecosystem health and public safety. Nevertheless, uneven adoption and adaptation among countries highlights ongoing challenges to fully achieving its objectives. Other relevant regulations cover machinery used for applying PPPs, classification and labelling, and the collection of statistical data on agricultural inputs [20,21,22].

#### 3.1.3. Evaluation of Regulatory Effectiveness and Improvement Initiatives

The effectiveness of regulations is evaluated regularly. After the review of Regulation (EC) No 1107/2009 within the framework of the European Commission’s Regulatory Fitness and Performance Programme (REFIT) [23], it was found that, despite progress, implementation of the regulation is still not satisfactory. Member States must do more to reduce dependence on chemical pesticides and improve transparency to increase trust in the system [24,25,26]. In response, the Commission proposed an amendment to the General Food Law, culminating in Regulation (EU) 2019/1381 [27] on transparency and sustainability of risk assessment, which was applicable from March 2021. These reforms reflect clear One Health objectives by aiming to reduce chemical-related risks, promote sustainable agricultural practices, and protect human, animal, and environmental health concurrently.

In parallel, several legislative proposals have been adopted to coordinate the hazard and risk assessments of EU chemicals that could have horizontal impacts on regulatory regimes, including REACH (Registration, Evaluation, Authorisation, and Restriction of Chemicals) [28] and regulations on biocides and plant protection products. As part of the EU’s “zero pollution”, a key commitment of the European Green Deal, on 14 October 2020, the European Commission published the Chemical Strategy for Sustainability (CSS) [29], under which the European Commission committed to publishing a proposal for REACH reform, with the aim of better protecting citizens and the environment and promoting innovation for safe and sustainable chemicals. However, despite progress in reducing some types of pollution, achieving the targets is still a challenge. The framework is still under development and all updates can be found on the European Commission’s dedicated website [30].

The Commission is also proposing to simplify current assessment methods, improve the quality and consistency of safety assessments across legislation and ensure that resources are used more efficiently to move to a ‘one substance, one assessment’ process for chemical safety assessments. Within this framework and in response to political and societal ambitions, EFSA has launched a strategic initiative called “European Partnership for Environmental Risk Assessment,” based on Next Generation Systems (PERA) [31], which aims for a more coherent and harmonised approach in the characterisation of the environmental risks of chemicals (starting with pesticides), given that there are many commonalities (e.g., common parameters in hazard assessment).

Critical issues in the application of the SUD [32,33,34,35] have also been highlighted, following European citizens’ initiatives [36] and parliamentary questions. As part of the European Green Deal and the Farm to Fork strategy [37,38], a proposal was made to revise the SUD into a Regulation on the Sustainable Use of Plant Protection Products (SUR). This proposal, which is currently on hold due to geopolitical reasons and economic concerns, aims to reduce the use and risk associated with chemical pesticides by 50 per cent by 2030, with monitoring through risk indicators and harmonised statistical data [39].

The European Green Deal prioritises sustainability with ambitious targets through the Farm to Fork and Biodiversity strategies, fostering resilient agroecosystems and minimising health risks holistically in alignment with One Health. However, practical socio-economic, agricultural, and policy constraints must be addressed for these goals to translate into consistent, measurable outcomes [40].

In conclusion, while these reforms are progressively aligned with One Health ambitions and provide a strong foundation for integrated health protection, further harmonisation, cross-sectoral collaboration, and adaptive governance are crucial to fully realise the One Health vision within EU PPP policy.

#### 3.1.4. Harmonised Risk Indicators and Data Criticality

Recently, two harmonised risk indicators (HRIs) have been introduced, based on the quantities of active substances placed on the market and the granted emergency authorisations. Regulation (EU) 2022/2379 and the subsequent Regulation (EU) 2023/1537 lay down the rules for the collection of harmonised statistical data on agricultural inputs, which have been mandatory since 2025 [41].

On 15 July 2025, the European Commission published updated progress towards pesticide reduction targets for the period 2011–2023 that shows an overall decrease in the use and risk of chemical pesticides and in the use of more hazardous pesticides (https://food.ec.europa.eu/plants/pesticides/sustainable-use-pesticides/pesticide-reduction-targets-progress/eu-trends_en, accessed on 1 November 2025). However, Article 15 of the regulation, concerning indicators, has been the subject of debate due to its ambiguity. An important criticism is that the indicators reflect the hazard rather than the actual risk, i.e., the combination of hazard and exposure, potentially misleading risk management decisions. This limitation can inadvertently encourage substitution with compounds that are less hazardous but not necessarily less risky—as in the case of neonicotinoids and their replacements. Indeed, these indicators provide a simplified metric and do not account for actual exposure levels, dose–response relationships, or the complex interactions that determine real-world risk. In a One Health context—where simultaneous consideration of human, animal, and environmental health is essential—relying solely on hazard-based approaches can result in an incomplete or misleading understanding of risks and limits the ability to realistically assess the effectiveness of mitigation measures, such as buffer strips for environmental protection or precision application techniques. Furthermore, data on actual pesticide use are not yet available at EU level but will be compulsorily available from 2028. Currently, harmonised data on pesticide sales have been collected since 2011 (Eurostat), which show an overall stability between 2011 and 2020, with Germany, France, Spain, and Italy as the main markets [42]. Despite the availability of these data, there remain limitations regarding completeness, level of detail, and national differences, which call for caution in the analysis [43]. Recent studies also indicate that global data on pesticide use are often incomplete and underestimated, with significant differences between countries [44].

Chemical risk assessment integrates hazard identification with exposure characterisation and dose–response analysis to estimate the likelihood and severity of adverse effects. This integrated approach enables a more accurate evaluation of potential impacts across interconnected health domains. The mismatch between hazard-based rankings and risk can challenge regulatory decisions, creating gaps in protecting ecosystems and public health effectively. Consequently, reliance on HRIs alone may compromise policy legitimacy and effectiveness, underscoring the need for more integrated risk metrics that better capture real-world outcomes. Therefore, advancing risk indicators that incorporate exposure data and interdisciplinary knowledge is critical for operationalising One Health principles in PPP regulation. This shift will enhance the ability to predict and mitigate combined human–environmental risks, fostering more informed, balanced, and protective management strategies [45].

To provide a clear overview of the key challenges and corresponding ongoing initiatives within the European regulatory and scientific framework for plant protection products, Table 1 summarises the major barriers to integrating One Health principles in risk assessment and governance, alongside current efforts to address these issues. This synthesis aids in framing the detailed discussion that follows on scientific methodologies, exposure assessment, communication strategies, and policy harmonisation.

Having established the legislative context, the following section delves into critical scientific challenges, focusing first on hazard identification and characterisation processes.

### 3.2. Critical Issues Related to Hazard Identification and Characterisation

Despite rigorous scientific evaluation reflected in EFSA’s detailed peer reviews of pesticide active substances such as glyphosate [46], several studies identify gaps in the data and the methodological limitations that impact comprehensive hazard characterisation. Missing information and residual concerns about low-dose effects, mixture toxicity, and long-term environmental exposure are frequently noted, underscoring the need for ongoing methodological advancements.

Risk assessment of PPPs is undergoing a transformation, due to the introduction of innovative methodologies, known as NAM (New Approach Methodologies). The following subsections reviews NAMs such as in vitro cell testing, in silico computational models, omics technologies, and artificial intelligence applications, which are designed to complement or replace traditional animal testing in risk assessment. European pilot initiatives showcasing practical progress and remaining challenges in NAMs integration will be presented, with examples from the Netherlands and the ongoing efforts by EFSA’s Partnership for Environmental Risk Assessment (PERA). In the following subsections, we examine regulatory and institutional barriers that hinder the uptake of NAMs, including the slow pace of regulatory acceptance, validation constraints, cultural resistance, and legal uncertainties.

#### 3.2.1. New Approach Methodologies (NAMs) Overview

These methodologies promise multiple advantages, including better relevance to human biology, higher mechanistic insights, reduction in animal use, cost-effectiveness, and faster data generation.

*In vitro and in silico methods:* In vitro tests on cell cultures (e.g., human hepatocytes) allow us to identify specific adverse effects, such as liver toxicity of new pesticides, reducing reliance on animal testing [47]. In parallel, QSAR (Quantitative Structure-Activity Relationship) models have been integrated by EFSA for the preliminary evaluation of active substances, demonstrating efficacy in predicting toxicological properties based on chemical structure [48].

*Omics technologies:* Metabolomics has revealed metabolic alterations in fish exposed to pesticides in European streams, providing biomarkers for ecotoxicological risk assessment [49]. Transcriptomics and proteomics studies, integrated into Adverse Outcome Pathways (AOPs), have elucidated toxicity mechanisms at the molecular level, as demonstrated in assessments of chronic exposure to neonicotinoids [50].

*Artificial intelligence:* EFSA pilot projects use machine learning algorithms to predict bioaccumulation and environmental persistence of pesticides, optimising preliminary screening [51]. These approaches analyse toxicological big data to identify complex patterns that are not detectable by traditional methods.

#### 3.2.2. European Case Studies

The afore-mentioned PERA (Partnership for Environmental Risk Assessment) project, coordinated by EFSA, promotes the harmonisation of NAMs in the environmental assessment of pesticides [52]. In the Netherlands, regulatory pilot projects have implemented in vitro tests and in silico models in authorisation procedures, reducing the use of animals by 30% in the last three years, as evidenced by Netherlands National Committee for the protection of animals used for scientific purposes (NCad) [53].

In the context of substances with limited data, the read-across approach, which integrates information from new approach methodologies (NAMs), is regarded as a valuable tool to minimise uncertainties and address data gaps in the risk assessment of chemicals in food and feed. The concept of read-across is based on the principle that structurally or mechanistically similar molecules tend to exhibit comparable properties. This methodology involves identifying data-rich (source) substances that closely resemble a data-poor (target) substance and using data from the source to predict the toxicity of the target. To ensure regulatory consistency and provide risk assessors and applicants with a clear framework for applying this approach, EFSA published comprehensive guidance in July 2025 [54], outlining systematic and transparent procedures for conducting read-across assessments.

#### 3.2.3. Regulatory Challenges

Current EU frameworks, such as Regulation (EC) No 1107/2009, mandate hazard identification primarily through established toxicological and ecotoxicological studies. While these frameworks set stringent standards, the continued reliance on animal testing and classical toxicology—despite recent innovations like New Approach Methodologies (NAMs)—slows adaptation and the integration of more holistic assessment approaches.

Regulatory inertia and the reliance on traditional hazard-based methods seems to limit the NAMs ability to capture the complex interactions between human, animal, and environmental health.

Zaunbrecher et al. [55] conducted a survey involving 1381 toxicologists, 75 per cent of whom were from North America, 15 per cent from Europe, and the remaining 10 per cent from other regions of the world, highlighting how regulatory acceptance represents a significant barrier to the adoption of alternative methods, such as in vitro and in silico.

This delay in adoption is largely attributed to the following:

*Rigidity and slowness of regulatory frameworks:* Traditional regulations are often based on established methods, such as live animal tests, which enjoy a long history of acceptance and validation. Changing these standards requires time, robust evidence, and a complex review process.

*Need for validation and harmonisation:* Regulatory authorities require that new methods demonstrate reliability, reproducibility, and biological relevance comparable or superior to traditional methods. This validation process is lengthy and costly, slowing down large-scale adoption.

*Cultural and institutional resistance:* Even among experts and decision-makers, there may be some mistrust of unconventional methods, especially when it comes to ensuring human and environmental safety, leading to a preference for ‘proven’ approaches.

*Legal and liability implications:* The use of methods that are not yet fully recognised may expose companies and regulators to legal risks or challenges, incentivising a cautious and conservative approach.

However, NAMs implementation/adoption in the evaluation process has been introduced, but for PPPs risk assessment, they should be seen as complementing rather than wholly replacing traditional methods, with careful consideration of their current gaps in human and environmental safety evaluation.

Indeed, despite their potential, NAMs have notable shortcomings in assessing human and environmental safety [56]. Critical complex endpoints like reproductive, developmental, neurotoxicity, carcinogenicity, and endocrine disruption effects remain challenging to fully capture with current NAMs. These methods are less able to completely replace animal testing when addressing systemic, multi-organ, or long-term effects, or accounting for population variability without additional supportive data. Regulatory agencies recognise that NAMs currently serve best as screening tools or hazard indicators, rather than sole sources of definitive risk conclusions. The use of NAM data may inform but not replace default safety adjustment factors in risk assessment paradigms to ensure protective outcomes.

In the context of pesticide risk assessment, NAMs are valuable for initial screening, mechanistic understanding, and supporting reduction in animal tests, potentially improving human relevance in safety conclusions. Yet, they must be applied critically by acknowledging their limitations, especially in complex toxicological endpoints and environmental interactions. Continuous efforts are underway to refine NAMs, harmonise data, and integrate emerging AI technologies to improve predictivity and regulatory acceptance while maintaining a precautionary approach toward human and environmental risk.

#### 3.2.4. The Challenge of Integration of New Approach Methodologies (NAMs) Within a One Health Framework

The integration of New Approach Methodologies (NAMs) within a One Health framework aligns well with the holistic perspective that One Health promotes, by reducing reliance on traditional animal testing and enhancing the specificity and relevance of safety assessments, contributing to more ethical and sustainable scientific practices and facilitating cross-sectoral data sharing and interdisciplinary collaboration, bridging gaps between environmental science, toxicology, and public health. The use of in vitro, in silico, omics, and AI technologies enables earlier identification of potential hazards that could affect not only humans but also wildlife and environmental health, fostering preventive actions and integrated risk management.

However, NAMs still require further validation to fully capture complex systemic effects and whether their adoption can help embody One Health principles by promoting safer, more predictive, and more humane approaches to chemical risk assessment that consider the health of all species and ecosystems in unison.

The following section addresses the equally complex issue of exposure characterisation, highlighting multiple exposure routes and the uncertainties that impact risk evaluation.

### 3.3. Critical Issues Related to Exposure Characterisation

This section discusses the various biological and environmental pathways through which humans and animals are exposed to plant protection products, emphasising the complexity of accurately estimating the exposure levels necessary for robust risk assessments.

Humans and animals may be exposed to plant protection products through different routes [57], such as diet, application areas, and the working environment. The main source of exposure for the general population is the diet, due to residues that are mainly on fruit and vegetables, but also on animal products and in drinking water. Occupational exposure occurs mainly on farms, during handling, the application of products, and maintenance of spraying equipment. People living near treated fields, such as residents or bystanders (e.g., cyclists), may also be exposed through drift and volatilisation of pesticides, as well as through contact with surface deposits. For children, exposure may also occur through contact with contaminated surfaces and the subsequent transfer from the hand to the mouth.

Estimating exposure to plant protection products is complex and presents many difficulties. The dose, frequency, duration, and route of exposure (e.g., inhalation, dermal, oral) are not always known or controlled and vary according to individual absorption and metabolism. The exposure level may consist of high doses for short periods or prolonged exposures at low doses, with acute effects appearing within hours or days, while chronic effects may emerge, even after years. Exposure assessment makes it possible, using indicators and models, to determine the level of exposure to the pesticide of non-target organisms and humans. Regarding the latter, different categories are considered, according to the main routes of exposure: consumer, operator, worker, and resident bystander. EFSA has developed a series of guidelines and technical reports that are continuously updated to support risk assessors, including methodologies for cumulative risk assessment (CRA) of dietary exposure to pesticide residues [58]. It is important to note, however, that CRA methodologies remain under development and are currently primarily applied in scientific and methodological contexts. While CRA represents a promising tool for better understanding combined exposure to multiple pesticide residues, it is not yet fully implemented for formal regulatory risk assessment purposes, due to ongoing challenges in methodology validation, data availability, and regulatory harmonisation. The extension of CRA into routine regulatory decision-making awaits further refinement and consensus among risk managers and assessors.

#### 3.3.1. Innovative Epidemiological Studies on Pesticide Exposure

Furthermore, behavioural factors and personal perceptions significantly influence the extent of exposure. Given the high uncertainty, the EU Regulation governing risk assessment for the placing on the market of active substances and formulations requires that where available, epidemiological studies conducted according to recognised standards must be submitted. However, many epidemiological studies on pesticide exposure and health effects have methodological limitations that undermine their use for assessment purposes. A systematic review of the epidemiological evidence conducted by EFSA [59] highlighted several shortcomings, which were also confirmed by the 2017 report of the EFSA Panel [60], which points out that the study of associations between pesticide exposure and health effects is more complex than in other areas of epidemiology. The main difficulties lie in (i) the large number of active substances on the market; (ii) the difficulty in accurately measuring exposure; and (iii) the frequent lack of quantitative and qualitative data on exposure to individual pesticides. In particular, poor exposure characterisation—i.e., the lack of detailed and direct assessments for specific pesticides—is the most significant limitation of most available studies.

To address these issues, EFSA recently published guidelines for the evaluation and integration of evidence from epidemiological studies, for use in scientific assessments [61]. These guidelines promote an approach based on the collection, evaluation, and analysis of scientific evidence (Weight of Evidence, WOE), which involves a structured process of identifying and critically selecting data from different sources (universities, research centres, industry, Eurostat, the scientific literature). WOE analysis includes critical evaluation of studies, data analysis, and transparency in the description of the procedures followed. When empirical evidence is limited, the Expert Knowledge Elicitation (EKE) method is used, following specific protocols and guidelines [62]. In summary, EFSA does not rely on a single study or type of evidence but considers the totality of available evidence to formulate robust and reliable scientific assessments.

#### 3.3.2. Innovative Approaches in Exposure and Effect Assessment

Following the identification and characterisation of hazards, it is crucial to advance innovative approaches in exposure and effect assessment. Better approaches need to be developed that not only include effect assessments but also integrate human biomonitoring (HBM), enhanced comparison of environmental monitoring data, exposure modelling, and evaluation of innovative exposure assessment methods. Assessing these aspects from a legislative perspective is very challenging, both in terms of cost and technical capacity. Overcoming these regulatory barriers is key to accelerating the transition to a more ethical, efficient, and sustainable risk assessment that reduces the use of laboratory animals and exploits the full potential of emerging technologies. This requires continuous dialogue between scientists, regulators, and stakeholders, investment in research and training, and the constant updating of guidelines and regulatory acceptance criteria. This is where the concept of partnership between risk assessors, authorities, and the scientific community can make a difference and help implement innovations in effect tests and exposure models and regulation [63].

We are therefore in a transition phase towards what is called Next Generation Risk Assessment (NGRA). The EU PARC project [64] can provide further insights. The project promotes the development and application of advanced methods, including in vitro, in silico, and integrated approaches based on biological data and computational models, as a continuation and extension of previous projects such as EU-ToxRisk [65] and HBM4EU [66]. The project is closely linked to the European Chemical Strategy for Sustainability and the Green Deal, aiming to better protect health and the environment while reducing the use of animal tests and integrating scientific innovation and regulatory dialogue.

The following section describes how risks are characterised, with an emphasis on cumulative and mixture effects that are relevant to One Health risk assessment.

### 3.4. Critical Issues Related to Risk Characterisation

The final step is the cumulative risk characterisation (CRA). Various methodologies have been formulated for the purpose of characterising the combined risk posed by PPPs that act in a similar way [67]. One such methodology involves the utilisation of reference values, such as health-based guidance values, for the determination of risk [68]. The concept of dose/concentration addition (DA/CA) involves the normalisation of the potency of each compound to an index compound (IC). Subsequently, to determine the potency of each component relative to the IC, the relative potency factor (RPF) for each compound is utilised to calculate the cumulative residue level of each sample, expressed as IC equivalents. The cumulative risk values for a given common toxic effect are then calculated and combined. The final step in the process is to characterise the risk, which includes describing the variability and the main sources of uncertainty. This approach facilitates the identification of risk contributors from PPPs and food commodities, as well as the affected subpopulations.

Another methodology is the Margin Of Exposure (MOE). The MOE is a measure of a chemical’s safety, used to compare its Point Of Departure (POD) and exposure, in which critical effect levels are compared to estimates of exposure, considering uncertainties in the available exposure and toxicological databases. Some regulatory bodies use ‘Margin of Safety’ (MOS) interchangeably with MOE, while others define it differently. To address inconsistencies in interpretation, and in line with the One Health approach, EFSA recently published a statement with the aim to standardise its terminology, establishing MOE as a primary metric for safety assessments across human and animal health evaluations [69].

For the PPPs mixture, the total MOE (MOE_T_) calculates the MOE for a group of chemicals by adding the reciprocal of individual chemicals’ MOEs and then taking its reciprocal [70]. Based on this approach and a regulatory threshold of MOE_T_, set at 100, EFSA completed several pilot dietary cumulative risk characterisations of pesticide residues, supported by an uncertainty analysis based on Expert Knowledge Elicitation (EKE).

One CRA considered the acute effects on the nervous system [71], while the other focused on two chronic effects on the thyroid system [72]. A third CRA considered the pesticides’ effects on cranio-facial alterations [73]. In all three CRAs, at least thirty-one sources of uncertainty affecting the input data, model assumptions, and assessment methods were identified.

Despite the considerable advancements in the CRA of multiple pesticide residues in food that have been made in the past decade, thanks to robust and up to date European diet consumption data that, together with harmonised residue levels monitoring system, allow consumer exposure estimation, the harmonisation of the CRA methods used by the different regulatory agencies is still a challenge. These include methodological difficulties in integrating diverse data across sectors, ongoing data gaps regarding environmental exposures and non-target species, and institutional silos that reduce consistent cross-domain interpretation. Addressing these barriers is essential for applying the MOE concept more effectively within an integrated One Health framework.

Conversely, no such level of information is available to characterise non-dietary exposure to pesticide mixtures in occupational or residential settings. Until now, in fact, no EU requirement has been in place to collect and monitor the agricultural use of PPPs. Consequently, data has only been collected by some Member States within the remit of their national regulations, making it difficult to retrieve. Only recently, the European Commission adopted an implementation of regulation No. 1107/2009, regarding the content and format of records of PPPs kept by professional users, which will only enter into force in 2026. As a result, methodologies or approaches to address non-dietary CRA are currently at the outset [74].

Building on risk characterisation, the following section highlights the essential role of effective risk communication in fostering transparency, trust, and public acceptance of risk management decisions.

### 3.5. Critical Issues Related to Risk Communication

The effective communication of risk is fundamental to bridging scientific assessment and societal perception, addressing behavioural, cultural, and socio-economic factors that influence the acceptance and implementation of mitigation measures.

Public perceptions of risk vary according to individual values and motivations, which can influence the acceptance of risk management outcomes, and risk communication plays a crucial role in ensuring a two-way dialogue between scientists, managers, and civil society, fostering transparency and trust in the decision-making process. It makes it possible to link the subjective perception of risk—influenced by cognitive and emotional factors—with the objective assessment, thus mitigating discrepancies that may generate unjustified fears or underestimations of real risks.

The human sciences have recognised that behaviour and attitudes are fundamental to proposing effective and acceptable mitigation measures [75]. In the context of the sustainable use of PPPs, it is essential to consider the socio-cultural aspects that influence the acceptance or rejection of technologies and innovations. When risk assessment results for pesticides are not effectively communicated to end-users and the wider community, the discrepancy between objective risk and perceived risk, which is influenced by human behaviour, can lead to poor adherence to the recommended good practices. Misunderstanding or lack of clear information can cause users to underestimate or overestimate risks, resulting in behaviours that deviate from safety guidelines. Therefore, transparent, accessible, and context-sensitive communication is essential to bridge this gap, ensuring, for example, that users not only comprehend the scientific assessments but also trust them enough to follow the prescribed measures that protect their health and the environment.

Proper communication can help to align perceptions with objective risks, promoting compliance and ultimately improving safe pesticide use.

The following two examples illustrate distinct aspects of the impact of risk communication and perception. The first addresses challenges related to the effective implementation and communication of mitigation measures, highlighting how discrepancies between scientific risk assessment and public understanding can hinder compliance. The second example focuses on the case of glyphosate, a widely studied molecule for which, despite extensive scientific data and institutional efforts, communication has fallen short in clearly conveying the health risks. This has resulted in public alarm and diminished trust in regulatory authorities.

These cases exemplify the critical distinction between measurable risk, where outcome probabilities are known, and uncertainty, where outcomes may be identified but their likelihood remains unclear. This distinction underscores the inherent difficulties in communicating complex risk assessments to the public and emphasises the need for transparent, nuanced communication strategies that acknowledge uncertainty and foster informed decision-making.

#### 3.5.1. Example 1—Exposure Management and Mitigation Measures

Risks identified through the risk assessment of PPPs can be effectively mitigated by implementing appropriate measures, which are closely linked to adherence to good agricultural practices. Failure to comply with these practices can lead to unintended exposures and environmental contamination, thereby undermining the outcomes of the risk assessment. Therefore, ensuring that users are fully aware of and comply with the label instructions is essential to safeguarding both worker safety and environmental health.

Recent studies show how economic and socio-cultural factors influence farmers’ decisions, which must balance safety, necessity, and cost. Some mandatory measures, such as buffer strips of considerable width, are difficult to implement in specific contexts, highlighting a ‘disconnect’ between those who define the rules (risk analysis) and those who enforce them (risk management) [76]. Moreover, the current Environmental Risk Assessment (ERA) framework lacks sufficient flexibility to accommodate scenarios for other types of applications (e.g., precision application techniques), active substances with particular properties (e.g., volatile chemicals), or for new technologies (e.g., RNAi). Application methods that can reduce environmental exposure, such as precision application technologies and drift reduction techniques, must be considered in the environmental risk assessment. For such scenarios, conducting an explicit problem formulation would help to develop a more fit-for-purpose environmental risk assessment [77]. A compendium listing conditions of use and mitigation measures for plant protection products was recently published [78], with the aim of reducing human and environmental exposure in the EU, providing a foundation for future mapping and technology validation.

#### 3.5.2. Example 2—The Case of Glyphosate and Uncertainty in Risk Assessment

Risk assessment for substances such as glyphosate inherently involves a degree of uncertainty, due to inconclusive or ambiguous scientific evidence. This uncertainty, however, should not be viewed as a weakness, but rather as an essential part of the scientific process that enhances the precision and clarity of data communication. Misunderstandings often arise because the public may interpret uncertainty as a lack of knowledge or reliability [79]. Additionally, individuals vary widely in how they perceive and weigh risks against potential benefits, influenced by personal values and motivations. While some are highly risk-averse, others may prioritise the potential benefits or even disregard risks altogether. Recognising and addressing these diverse perspectives is crucial for effective communication and the acceptance of risk assessment outcomes [80].

Risk-benefit assessment (RBA) is a multidisciplinary methodology requiring a wide range of expertise and collaboration between scientific disciplines (chemistry, nutrition, toxicology, microbiology, mathematical epidemiology, statistics, etc.), which is applicable to different fields of analysis.

This aspect is taken into account by EFSA, who have recently updated the Dietary Reference Guideline, which focuses on nutrition in relation to human health and considers the risks arising from the presence of hazards in food, weighing them against the benefits derived from diet, food and/or food components [80] in detail. The perception of both risk and potential benefit is one of the elements taken into account. Several factors are reported to have a negative influence on risk perception, including a lack of confidence in communications and data provided by the industry and the order in which information is provided.

Public perception of the risk assessment of PPPs within the European Union exhibits considerable variability. As reported in the Science Advice for Policy by European Academies (SAPEA) report [81], for instance, in 2013, EFSA assessed the risks posed by neonicotinoid pesticides—specifically clothianidin, imidacloprid, and thiamethoxam—to bee populations. Based on evidence identifying these substances as harmful to European honeybees, EFSA recommended restricting their use in PPPs. This decision was broadly accepted by the public. Conversely, EFSA’s assessment of glyphosate, following a similar scientific approach, concluded that it is unlikely to be carcinogenic to humans, although small uncertainties remain. Despite transparent communication of this conclusion, the public response was overwhelmingly negative, sparking intense debate. This reaction appears to be unique against the backdrop of more than 8000 assessments published by EFSA over the past 15 years and remains difficult to rationalise fully.

Further complicating public understanding were the contrasting hazard classifications issued by organisations such as the International Agency for Research on Cancer (IARC), which relied exclusively on publicly available data subjects to independent scientific review [82]. These divergent evaluations contributed to confusion and calls from public initiatives to ban glyphosate-based herbicides, citing their links to human cancer and environmental degradation. Such initiatives also advocate for stricter regulatory policies, including the exclusive use of studies commissioned by public authorities, rather than industry stakeholders.

For glyphosate’s assessment from 2019 to 2023, 2400 studies were used, more than 400 comments from public consultations were accepted, and 27 national authorities and more than 90 experts were involved. The glyphosate dossier is two to four times larger than a normal dossier produced for the authorisation application procedure. On 28 November 2023, the European Commission renewed the approval of glyphosate for 10 years. This is in line with EU legislation that obliges the Commission to adopt an implementing regulation when there is no qualified majority, for or against, in the standing and appeal committee, as in the case of glyphosate. In favour of public initiatives, it was evident that EFSA has interacted even more with stakeholders, and the transparency of data and information has increased, also thanks to the implementation of the Transparency Regulation (EU) 2019/1381 [83]. Furthermore, more studies have shown that the presence of AMPA (amino-methylphosphonic acid), the main transformation product of glyphosate, is related to other non-agricultural activities, such as weed control for urban purposes, but especially wastewater treatment plants. In fact, AMPA is known to be a transformation product not only of glyphosate, but also of aminopolyphosphonates used in many applications, including laundry detergents. A recent meta-analysis on glyphosate concentrations in river waters in the US and Europe points to the possibility that the main source of glyphosate in Europe may not be herbicide applications, but rather wastewater [84]. This is supported by the comparison of seasonal patterns, constant concentration data even in the absence of agricultural applications, and the presence of glyphosate and AMPA in wastewater treatment plants. Applied mitigation strategies that include restriction of even urban use have not reduced concentrations in surface water, and the concentration patterns of AMPA and glyphosate are strikingly similar, despite the different input pathways. These results indicate the need to review the sources and management strategies for glyphosate in European waters.

#### 3.5.3. One Health Approach and PPPs Public Perception of Risk

Balancing the requirements of chemical risk assessment, therefore, with the social expectations of risk analysis is, in an OH approach, one of the main challenges to be faced for PPPs.

In addition, we must take into account the subjectivity that is inherent in chemical risk assessment, often termed “intuitive toxicology,” that has been recognised in the literature since at least 1992 through the pioneering studies of Klaus and Slovic [85]. This concept highlights a natural element of uncertainty stemming from varied interpretations and perceptions of risk among toxicologists, which are influenced by differences in their professional backgrounds—such as regulation, industry, and academia—as well as factors like gender and worldview. In fact, the study found considerable divergence among toxicologists: 41% agreed or strongly agreed with the statement that “if a scientific study produces evidence that a chemical causes cancer in animals, then we can be reasonably certain that it will cause cancer in humans,” whereas 58% disagreed or strongly disagreed (with 1% uncertain or neutral). Although scientific methodologies necessarily involve a degree of subjectivity—through assumptions and interpretations, particularly in extrapolation—disagreements among experts on core toxicological principles significantly affect both the perception and credibility of risk assessment results. Such expert disagreements can strongly influence public confidence, often heightening concerns around chemical risks [86], undermining trust in scientific expertise, and complicating public discourse—phenomena vividly illustrated by the controversy surrounding the herbicide glyphosate.

In alignment with the One Health approach, effectively addressing the public perception of risk requires more than scientific risk assessment alone. One Health promotes interdisciplinary collaboration and active engagement among diverse stakeholders, including policymakers, farmers, local communities, and environmental experts, to collectively pursue shared goals of health and sustainability. By integrating local knowledge and socio-cultural factors through participatory methods, understanding and acceptance of risk assessments are improved, thereby enhancing the efficacy of risk management strategies [87]. Such collaborative efforts support the development of sustainable and resilient agricultural systems, embodying the core One Health objective of optimising health outcomes across the human, animal, and ecosystem domains.

Following communication challenges, Section 3.6 discusses subjective components influencing chemical risk assessment and the critical need to rebuild public and stakeholder confidence.

### 3.6. Subjective Components in Chemical Risk Assessment and the Need to (Re)Build Confidence in the Assessment Process

This final section highlights the subjective nature of risk interpretation among both professionals and the public, underscoring transparency, trust, and stakeholder engagement as fundamental pillars of effective risk governance aligned with One Health principles.

Tensions between industry innovation, regulatory conservatism, and public distrust complicate the implementation of One Health principles in PPP regulation. Regulatory frameworks often lag behind scientific advances, which can slow the adoption of innovations when being included or considered in the panorama of mitigation measures. Meanwhile, public mistrust stemming from perceived opacity or conflicts of interest challenges the effectiveness of risk communication and compliance. Addressing these tensions requires fostering transparent, participatory governance models and interdisciplinary collaboration.

The examples previously discussed emphasise that public trust in the risk assessment process and the expertise involved is a critical determinant of acceptance of the information produced and the perceived balance between risks and benefits. Unfortunately, trust is fragile and often easier to erode than to build, with recent scientific disagreements regarding risk severity tending to undermine public confidence.

Focusing here on the social and cognitive dimensions:

- Risk perception plays a vital role in societal risk management and strongly influences how uncertainty and ambiguity are addressed [88].
- Transformation within these processes requires trust, which in turn depends on transparency—ensuring stakeholders can understand system functioning, identify weaknesses, and see how these are or can be addressed [89].
- Transparency must be coupled with a shared commitment among diverse stakeholders who align on common visions and values that are central to decision-making and strategy [89].
- Behavioural and cultural factors are essential to the exposure assessment and risk management of plant protection products [90].
- Clear communication of uncertainties inherent in exposure and effect assessment is paramount [91].

Table 2 summarises key challenges identified within the EU regulatory and scientific landscape for PPPs and highlights the ongoing initiatives and efforts documented in the paper to address these challenges. The aim is to provide a clear overview of the multifaceted barriers and corresponding opportunities for advancing integrated One Health approaches in risk assessment, governance, and communication.

## 4. Discussion

The regulation of Plant Protection Products (PPPs) in the European Union is situated within a broader chemical food safety framework that is increasingly evolving toward integrated approaches. These products hold a crucial role in the EU’s food law and Chemical Food Safety framework and are essential for crop protection, ensuring food availability and quality. The interrelation with the One Health approach is increasingly recognised: pesticide management is not only about chemical safety in food but also about protecting environmental health and the interconnected human–animal–ecosystem health interface. One Health encourages integrated risk assessment and regulation that accounts for cumulative exposures, environmental impacts such as biodiversity loss, and indirect effects on human health. The collaboration of EU bodies such as EFSA, ECHA, and ECDC under One Health frameworks aims to operationalise this holistic perspective, enhancing the capacity to anticipate and mitigate combined risks from pesticides, thereby harmonising chemical safety, environmental protection, and public health goals in a sustainable way [92]. But, although the EU has a robust regulatory framework for PPPs, our analysis shows that improvements to the overall process are needed to achieve the Green Deal’s ambitious goals.

PPPs are regulated within the EU food safety framework to ensure human, consumer, and non-target organism protection from harmful residues, while the One Health approach fosters the enhanced integration of environmental and health risk assessment, promoting more comprehensive and sustainable pesticide management. To date, the “One Health” perspective has primarily emphasised the interconnectedness of environmental, animal, and human health, with relatively less focus on PPPs, integrated pest management, and agricultural practices, as evidenced by Falkenberg T et al. (2022) [8]. These latter aspects, which are inherently linked to plant health, represent a complex yet indispensable challenge for effectively safeguarding both public health and the environment.

Building on this foundation are the following:

- Current regulatory systems for risk assessment face several challenges that must be addressed. These include the need to evaluate the cumulative risk of an increasing variety of chemicals and mixtures, the complexity of assessing diverse health effects such as endocrine disruption and neurotoxicity, and the emergence of new materials like nanoparticles in product formulations.
- To address ethical and economic concerns, innovative methods [47,48,49,50,51]—including in vivo and in silico methods (e.g., quantitative structure–activity relationships, omics technologies, artificial intelligence, and automated processes)—have also been adopted for PPPs in toxicological assessments. While these advances enhance evaluation, they also demand new skills from assessors, increase the decision-making complexity, and introduce uncertainty, all of which impact the credibility of the process.
- Ethical considerations are central to this evolution. The reduction demand in animal testing and the adoption of more humane, transparent, and precautionary approaches is aligned with the principles of sustainability and One Health [93].
- To meet the ambitious EU objectives, the role of exposure science must also be strengthened. Currently, pesticide risk assessment in Europe is mainly hazard-driven, although guidelines and tools for chemical exposure assessment have evolved to meet the needs of specific policy areas.
- Expert disagreement and scientific subjectivity influence perceptions of risk assessment credibility, particularly when inconsistent interpretations raise public concerns, as exemplified by controversies such as glyphosate, presented in Section 3.5.2 Example 2, for the case of glyphosate and uncertainty in risk assessment.
- Public trust plays a pivotal role: confidence in science reduces perceived risks and boosts perceived benefits, while distrust in industry or conflicting information heightens risk perception. Confusion, conflicting messages, or lack of knowledge further amplify risk concerns. Although PPPs undergo rigorous risk evaluation, public opinions often emphasise environmental and health risks more than the benefits related to food safety and security [82,83].
- To meet the EU’s ambitious risk reduction targets, it is essential to assess the effectiveness of mitigation measures, including the potential impact of new technologies and innovations. Improving how the effectiveness of these measures is addressed and calculated in specific contexts could help to minimise human and environmental exposure to PPPs.
- To promote the effective use of PPPs, enhance communication, and bolster confidence in the entire risk assessment process, it is imperative to examine the behavioural and cultural factors, as well as risk perception assessments at contextual and territorial levels. Involving qualified professionals from the agricultural, forestry, livestock, and environmental sectors is essential to improve policy implementation and ensure an integrated approach according to the “One Health” principles and the UN Sustainable Development Goals. Agronomists, farmers, and rural communities play a pivotal role in managing sustainable agricultural practices and safeguarding the environment and rural livelihoods [81]. Their participation is vital to the effectiveness of environmental policies and crucial to preventing rural depopulation and the higher social and environmental costs associated with land management, as highlighted in the most recent paper of Vos et al. (2025) [44], addressing the unresolved challenges between the “One Health” approach and the agricultural sector.

In summary, an objective risk assessment of PPPs requires greater multidisciplinary integration, as well as the continuous updating of methodologies to reflect new scientific evidence and meet the requirements of public health and environmental protection.

A “One Health” approach, which fosters dialogue between science, policy, and civil society, is essential to overcoming the current critical issues and promoting more sustainable and resilient agricultural systems. Collaboration among key EU agencies exemplified by the Cross-Agency One Health Task Force reflects a commitment to operationalise One Health in regulatory science. This joint framework fosters strategic coordination, research synergy, and capacity-building to streamline scientific advice and risk assessments across sectors, underpinning more harmonised, transparent, and sustainable regulatory policies. Consequently, the role of PPPs within the EU chemical food safety framework is progressively oriented toward serving as a vital component of an integrated One Health approach, which is essential for a resilient and balanced protection of global health.

## 5. Conclusions

This article presents a perspective on the evolving integration of the One Health framework into the European regulatory landscape for PPPs. While grounded in a thorough review of the scientific literature, regulatory policies, and emerging methodologies, the primary aim is to provide a critical interpretative outlook, rather than a conventional systematic review. This perspective fosters a holistic understanding of the interplay between human, animal, and environmental health in the context of chemical risk assessment and regulation.

The study acknowledges the inherent limitations: notably, the reliance on existing data and public policy documents without original experimental research. Furthermore, the rapidly changing regulatory environment and scientific advancements necessitate continuous updates to maintain relevance. Despite these constraints, this perspective offers valuable insights by synthesising information streams and highlighting emerging challenges and opportunities from a multidisciplinary standpoint.

The strength of the One Health approach lies in its integrative and collaborative ethos, promoting cross-sectoral partnerships and inclusive stakeholder engagement. Applying this framework to plant protection product regulation enables more robust, harmonised strategies that simultaneously safeguard human health, animal welfare, and environmental integrity. This is especially critical in addressing complex issues such as chemical mixtures, climate change impacts, and societal expectations regarding transparency and risk communication. To facilitate effective One Health implementation, the establishment of pilot programmes, as proposed at the EU level, is vital. Such initiatives enable the capture of complex interactions across human, animal, plant, and ecosystem health, thereby guiding adaptive management. Governance models must emphasise inclusivity, transparency, and active stakeholder participation to build trust and support dynamic decision-making. Additionally, revising PPP authorisation criteria to explicitly incorporate ecosystem services and sustainability principles is essential for fully aligning regulatory outcomes with the One Health objectives. The Sri Lanka case serves not only as a cautionary example but also as a lens through which to analyse equity, trade dependencies, and disparities in technological access in global pesticide governance. A comprehensive One Health perspective must incorporate global justice dimensions, recognising differential impacts on vulnerable populations and the necessity for equitable access to sustainable technologies and support systems. This is particularly critical, given the transboundary nature of pesticide environmental and health impacts.

Ultimately, this perspective supports strategic innovation in risk assessment methodologies and governance practices, underscoring the indispensable role of the One Health principles. Embracing this approach is essential for advancing sustainable, resilient agricultural systems and comprehensive health protection policies at the European level and beyond.

## Figures and Tables

**Table 1:** Key challenges and ongoing initiatives in EU plant protection product risk assessment, aligned with One Health principles.

Challenge	Ongoing/Emerging Initiatives
Cumulative Risk Assessment	Development of cumulative risk assessment methodologies; EFSA guidance on dietary exposure (CRA); refinement techniques such as Expert Knowledge Elicitation (EKE) for uncertainty analysis
Exposure Characterisation	Continuous updates by EFSA on exposure assessment methods; harmonisation of data collection related to exposure sources and vulnerable populations
Adoption of NAMs (New Approach Methodologies)	EFSA’s Partnership for Environmental Risk Assessment (PERA); implementation of in vitro and in silico assays; omics and AI technologies; European pilot projects; regulatory validation challenges
Public Trust and Communication	Regulation (EU) 2019/1381 enhancing transparency; inclusive stakeholder engagement; risk communication strategies to build trust and manage risk perception
Methodological Complexity and Data Gaps	Initiatives for harmonised scientific assessments; simplification efforts such as “one substance, one assessment”; continuous updating of evaluation methodologies aligned with evidence

**Table 2:** Challenges and opportunities in integrating One Health principles into Plant Protection Product risk assessment.

Key Point	Brief Description	Challenge for One Health Integration
Complex Legislative Framework	The EU has a robust, multilayered legislative framework protecting human, animal, and environmental health through regulations and directives for PPPs. Directive 2009/128/EC promotes integrated pest management and pesticide reduction, balancing productivity and sustainability.	Regulatory inertia slows cross-sector coordination, hindering integrated One Health responses and limiting advancements in agricultural practice.
Harmonised Risk Indicators	Harmonised risk indicators and data collections rely on hazard-based metrics that miss real exposure and risks.	Hazard-based focus limits capturing complex human–environment interactions that are essential to understanding One Health impacts.
Innovative Methodologies (NAMs)	New approaches (in vitro, in silico, omics, AI) promise better hazard identification and reduced animal testing but require validation.	Slow regulatory acceptance and validation delay the integration of innovative science that is necessary for One Health.
Exposure Characterisation	PPP exposure pathways are complex with uncertainties, requiring improved cumulative risk assessment methods.	Scientific and data gaps hamper accurate, holistic exposure assessment across human, animal, and environmental health domains.
Toward One Health	Legislative and scientific landscapes are evolving toward One Health, requiring multidisciplinary collaboration and adaptive governance.	Institutional silos, resource constraints, and policy fragmentation impede fully integrated transdisciplinary One Health action.
Communication	Clear and accessible communication supports the One Health approach, enhancing awareness, collaboration, and transparency in PPP risk assessment.	Effective, transparent communication across diverse stakeholders remains challenging, especially in conveying scientific findings to foster trust and coordinated action in One Health contexts.

## Data Availability

No new data were created or analysed in this study.
